# Contract Law When the Poor Pay More

**DOI:** 10.1093/ojls/gqae002

**Published:** 2024-02-19

**Authors:** Joseph Spooner

**Keywords:** contract law, consumer law, adjudication, equality, distributive justice

## Abstract

Taking inequality as a key challenge of our time, this article aims to highlight consumer markets, and their underpinning legal ground rules, as important contributors to inequitable wealth distributions. It illustrates how product design, as manifested in contractual terms, can allow firms to evade competition and divert resources upwards along society’s wealth distribution curve. It then highlights the contestable legality of certain pricing practices, such as ‘contingent charges’, and the challenge they pose to fundamental principles of contract law. An in-depth view of the 2015 case of *Beavis v ParkingEye* argues that the UK Supreme Court has validated contingent pricing models in a manner unsupported by traditional contractual reasoning and unjustified by contemporary market failure analysis. The article asks contract law to confront the reality that it shapes market distributions in economically and politically significant ways, and appeals for greater scrutiny of the contribution of contract law adjudication to inequality.

## 1. Introduction: How Contract Law Adjudication Underpins Market-Driven Inequality

As we have slouched from crisis to crisis over the past 15 years, it is hard not to hold a sense that ‘something is wrong’ with our contemporary economic order of financialised capitalism.^1^ A core critique has emerged regarding the tendency of modern capitalism towards unequal distributions of resources,^2^ to the point at which inequality has been declared the ‘defining challenge of our time’.^3^ The Global Financial Crisis and Great Recession taught us the negative macroeconomic effects of distributing resources away from low- and middle-income households (society’s chief spenders),^4^ tearing apart old ideas regarding the equity–efficiency trade-off.^5^ Rather than endangering prior economic trends, the shock of COVID-19 embedded them further, often exposing existing inequalities.^6^ The post-pandemic cost-of-living crisis brought into sharp relief the incongruity between rising corporate profits and declining household living standards.^7^ These latter trends perhaps illustrate the overlooked contribution of consumer markets to contemporary inequality. Attention has long focused on the more obvious dynamics of inequality in employer–worker conflict and in austerity-coloured tax-and-transfer politics.^8^ It is now increasingly clear, however, that victories and defeats in these latter arenas lose meaning if businesses can extract excess value from consumers in the emerging site of class conflict represented by mass retail markets.^9^ A sense seems to prevail that current economic conditions indeed facilitate this value extraction, with markets characterised by ‘wealth transfers instead of wealth creation’.^10^

One facet of this wealth transfer is the way in which product design, as manifested in contractual terms, can allow firms to evade competition and divert resources upwards along society’s wealth distribution curve. Under a process of ‘seduction by contract’, contemporary firms use knowledge of consumer psychology and behaviour to design contracts ‘to maximise not the *true* (net) benefit from their product, but the (net) benefit *as perceived by the imperfectly rational consumer*’.^11^ This means that in modern markets contracts are characterised by complexity and design features which defer and/or conceal future costs in order to make an offer immediately more attractive.^12^ This phenomenon is encapsulated in the description, now widely used in the United States by the Biden administration, of ‘junk fees’—‘unfair or deceptive fees that are charged for goods or services that have little or no added value to the consumer’.^13^ This article focuses on the legal underpinning of one subset of such junk fees—what one could call ‘contingent charges’.^14^ The specific practice of contingent charging holds particular relevance due to its widespread prevalence throughout the economy, the manner in which it confronts fundamental assumptions of contract law and the consequent challenge posed by recent and well-known UK Supreme Court cases—*Beavis v ParkingEye*^15^ and its prior sister case of *Abbey National v Office of Fair Trading*.^16^

This article first aims to illustrate how product design, embodied in contractual terms, enables businesses to shirk the constraints of market competition and re-route resources from households to the holders of corporate wealth (section 2). While the harm to individual consumers of product design features such as contingent charges are reasonably well recognised, the article seeks to highlight the underappreciated way in which the aggregate effect of such market practices may exacerbate inequalities. Section 3 discusses how legal ground rules underpin all markets, meaning that regressive distribution through market mechanisms depends on the legal enforceability of extractive product designs. This leads to recognition of the sometimes overlooked distributional stakes of contract law adjudication and the realisation that our society places considerable distributive powers in the hands of judges who must decide upon the legality of new products and business practices. The article presents the Supreme Court case in *ParkingEye* as a decision with ramifications well beyond the two parties to an individual dispute. The decision set the ground rules for a billion-pound market, while also determining the possibility of legal challenge to a regressive contingent pricing model which prevails across the contemporary economy (section 4). The Supreme Court ultimately legitimated contract design practices which appear to run counter to classical theoretical ideas of contract law and conflict with contemporary regulatory thinking. The article argues that the Court, in deference to the business practices of market incumbents, contorted important contract law rules against penalties and unfair terms.

The penalty doctrine establishes that a contract drafter can place a counterpart under an obligation to pay a specified sum in the event of her breach of contract only if this sum is not excessive. Unfair terms legislation in turn invalidates certain one-sided clauses concealed in contractual ‘fine print’. When first required to consider the potential application of these doctrines to contingent charges, the Court placed a wide range of contractual clauses and pricing practices beyond legal review and into untouchable categories of ‘primary obligations’ or ‘price terms’. In reaching this position partly in reliance on the scale of revenues produced by such charges, the Court created a paradoxical position under which the greater a business’s income stream generated by these suspect pricing practices, the lower the likelihood of court review of the practices’ legality (section 5). Moving to the question of when a reviewable clause might be determined unfair or excessive, the judges effectively rewrote the penalty doctrine and ‘watered down’^17^ unfair terms legislation in a manner which appointed themselves as arbiters of the ‘legitimacy’ of business models (section 6). The Supreme Court then proceeded to legitimate business models under which firms depend for profit on unexpected outcomes detrimental to customers, thus misaligning the incentives of contracting parties and compromising the idea that contracts are founded in mutual benefit (section 7). Finally, faith in the efficiency of extant business practices led the court to establish market prices as the only measure of the proportionality or fairness of contractual charges, an approach singularly inappropriate to confront contemporary challenges of sector-wide market failures (section 8).

Certain technical features of contract law may lack much political or cultural salience, buttressing a viewpoint that doctrine carries little distributive import.^18^ The principles applied to contingent charges in the *ParkingEye* decision—the penalty doctrine and unfair terms rules—arguably make distributional consequences more visible. The penalty doctrine exists to guard against contractual traps and exploitation; and concerns the stuff of Shakespearean high drama and pounds of flesh. Similarly, unfair terms legislation can target the junk fees whose eradication is now a sufficiently popular cause to feature in books of Pulitzer Prize-winning poverty abolitionists^19^ and State of the Union addresses.^20^ These contractual doctrines are both distributionally consequential and ‘morally gripping’,^21^ making it difficult to argue that they are apolitical technical rules. By focusing on these issues, the article asks contract lawyers and judges to confront the reality that decisions in this sphere shape market distributions in economically and politically significant ways, and so appeals for greater scrutiny of the contribution of contract law adjudication to inequality.

## 2. Market Failure and Distribution

### A. An Equilibrium of Manipulation and Distortion: Contingent Charges and Inequality Beyond the Car Park

The dominant contemporary mode of legal and regulatory market analysis is the ‘market failure’ framework.^22^ It assumes that in the ideal of an efficient market, rational choices of ‘sovereign’ consumers drive producers to offer value under the disciplining pressure of free competition.^23^ Traditional market failure analysis justifies regulatory ‘intervention’ when the conditions for efficient private ordering are absent—where market conditions show imperfect competition, information asymmetries, barriers to entry or exit, significant transaction costs and/or externalities (social costs).^24^ Behavioural economics adds further well-established grounds for regulation, showing how features of human decision-making mean that our choices routinely depart from those of the *homo economicus* ideal of rational consumer choice—we demonstrate behaviours such as *optimism bias* and *time-inconsistent preferences*, and reach conflicting decisions where identical choices are framed differently. Firms competing for profit must design products to exploit these ‘irrationalities’. This produces ‘an economic equilibrium that is highly suitable for economic enterprises that manipulate or distort our judgment’,^25^ causing us to overpay for goods which do not provide commensurate value.^26^

Contingent charges are examples of market practices based on complex contract designs and pricing structures, which ‘increase profits at the expense of consumers’.^27^ Generally, competitive pressures create limits to the headline prices which firms can charge. Accordingly, firms hold incentives to advertise low headline prices prominently, only to drive revenue through less visible—and so, less competitive—fees and charges, under practices described as ‘price shrouding’.^28^ One such practice is the use of contingent charges—charges which only become payable on the (non-)occurrence of certain outcomes which are largely unforeseen, undesired, unpredicted or poorly understood by consumers. These contingent charges may be invisible to consumers due to informational asymmetries under a ‘market for lemons’ equilibrium,^29^ as consumers do not read relevant standard form terms.^30^ Additionally, even in the unlikely event of consumers holding all relevant product information, cognitive biases mean that consumer evaluations of the likelihood of contingencies arising will depart wildly from the standard of economic rationality. Consumers’ ‘time-inconsistent preferences’ mean that we will be attracted by low introductory offers, even where a product works out more expensive in the long run.^31^ Our ‘optimism bias’ causes us to discount unduly the possibility of adverse events or costly contingencies.^32^ This is not even to consider that avoidance of relevant contingencies may lie outside a consumer’s control—a consumer’s ability to perform a contract may be dependent on external shocks and ‘life accidents’ such as employment loss, ill health or relationship breakdown.^33^ Developments in behavioural research allow firms to design products and advertising with the very aim of exploiting these departures from economic rationality.^34^ Progress in credit-scoring systems, algorithmic decision making and machine learning facilitate these processes, as firms can accumulate vast amounts of user data, customising products, predicting accurately which contingencies are likely to impact sizeable customer groups and marketing for maximum profit.^35^ Competitive pressures between firms then mean that once these methods of exploiting consumer error are developed, they quickly spread and flourish across markets—no firm can afford to cede market share to its competitors and leave ‘money on the table’. The result is an equilibrium where manipulation or distortion of consumer decisions becomes the norm across a market.^36^

Firms are all too aware that the reality of product use departs significantly from consumer expectations, and so their use of concealed contingent pricing ‘reveal[s] a calculated business strategy to use complex contracts to extract additional charges from consumers’.^37^ These strategies extend well beyond the familiar example of firms offering us a free trial of a subscription service, knowing that our human inertia will prevent us from terminating the subscription before we begin to generate monthly revenue for the firm whether the service is valued or not.^38^ Credit card lenders know that most consumers do not even consider interest rates and default charges when shopping around, as they expect to repay their credit card balances on time.^39^ This creates strong incentives for lenders to respond to systemic consumer error by selling to higher-risk clients who are likely to fail in making monthly repayments,^40^ and so fall into a ‘sweatbox’ of continuous monthly fees and interest payments.^41^ Default, arrears and ‘persistent debt’ are profitable. Similarly, in the billion-pound market for bank account overdrafts, banks have earned outsized revenues from the ‘unarranged overdraft’.^42^ This is a euphemism for the market practice under which, rather than refusing to honour payment requests when customers hold insufficient funds, banks fund the payment by automatically extending high-cost credit, with charges and interest regularly exceeding a 10–20% effective *daily* interest rate.^43^ Consumers shopping for the contemporary necessity of a bank account do not tend to understand that an overdraft facility is debt and many who end up borrowing at high cost are even unaware that they are using the facility.^44^ In at least 50% of cases, recourse to unarranged overdrafts makes no economic sense since the relevant users have cash or cheaper credit lines available to make the relevant payment.^45^

Similarly, if all consumers used high-cost, short-term credit—‘payday loans’—in the manner advertised, as an expensive (sometimes approaching 4000% APR) but rational solution to an urgent and exceptional once-off emergency, lenders would *lose* money on each transaction.^46^ The real money (50% of revenue) in this market—which enjoyed explosive growth before Financial Conduct Authority (FCA) regulatory measures curbed its excesses—arises from ‘rolling over’ borrowers who were unable to pay their initial loan and retaining this customer long beyond payday.^47^ Rather than using this last-resort finance to cover a single emergency, the average consumer takes six loans.^48^ While the headline high interest rates in this market quickly became widely recognised, there has been less understanding of the ‘absolute dependence’ of business models on contingent revenue,^49^ generated by the recurrence—inevitable to lenders, but unforeseen by many borrowers—of the financial difficulty that triggered a consumer’s initial loan.^50^

Business models involving low headline prices combined with ‘shrouded’ contingent charges are prevalent in our economy, making the Supreme Court cases discussed below significant beyond the specific markets in which they arise. Other examples abound,^51^ under economic conditions in which widespread financialisation has seen even basic consumer purchases transformed into speculative exercises of risk management. Universal ‘Spotification’ has made the spot transaction increasingly rare.^52^ Across consumer markets, the product being sold is often a complex contract,^53^ with the true financial costs dependent on calculations of the likelihood of certain contingencies arising. Under this gamble, the odds are stacked in favour of the corporate sector which designs these contracts.

### B. Aggregate Effects of Market Failures: Redistributing Resources from Consumers to the Corporate Sector

Dominant market failure analysis and related law-and-economics frameworks are known for a tendency to disregard distributional and macroeconomic issues.^54^ Despite a willingness to accept that markets regularly depart from idealised efficiency, cause harm to individual consumers and require regulation, these perspectives are less prepared to acknowledge the aggregate distributive effect of widespread market failures.^55^ A disinclination to explore distributive issues may also arise from the view that regulatory efforts at redistribution may be futile, since ‘it is well understood’ that ‘prices generally adjust to reflect the cost of legal rules’.^56^

Where inequality in market outcomes are acknowledged, they are often discussed in terms of an equity–efficiency trade-off^57^ and an inequality *between* consumer groups, whereby cross-subsidisation arises under an equilibrium in which a (generally higher-income) majority benefits from lower prices while lower-income groups pay the highest costs.^58^ Indeed, regulators repeatedly find that pricing methods exploiting consumer irrationality often lead to the highest costs falling on lower-income groups.^59^ High-cost credit products based on the ‘rollover’ model of revenue generation are widely understood as being disproportionately used by low-income groups.^60^ Just 3% of arranged overdraft borrowers pay half of all fees; and for unarranged overdrafts, an even smaller group of just 1.5% of customers produce more than 50% of firms’ fees.^61^ For unarranged overdrafts, this small group of customers is 70% more likely to be drawn from deprived areas, lower-income households and Black, Asian and minority ethnic communities.^62^ Even in markets not directly targeted at low-income groups, such as the parking sector under review in *ParkingEye*, contingent charges can have disparate outcomes for high-income consumers compared to those of lower-income groups. Irrespective of theories of attention scarcity linked to the experience of poverty,^63^ the inherent regressivity of flat-rate contingent charges disproportionately impacts low-income groups.^64^ While wealthier consumers might view an unexpected charge as an annoyance, the liquidity constraints of the poor can quickly see this additional cost become a serious problem—a default which grows as debt collection fees are added, and which may then transform into a negative record on the debtor’s credit history, further condemning them to a spiral of sub-prime, high-cost market access.^65^

This view of *intra-consumer* inequality is, however, only part of the picture of market-driven inequality. A perspective which considers that higher-income consumers inevitably pay less when the poor pay more risks pitting consumer groups against one another while concealing questions of corporate profits. Corporate gains from overcharging low-income consumers can only benefit higher-income groups if one assumes unrealistically such intense competition that every penny raised from the overcharged minority is being passed on to the majority group of customers in discounted prices. Such a view ignores how contract design which exploits consumer error can allow firms to extract supra-competitive prices^66^ across all consumer groups.^67^ Regulation may indeed cost firms, but where it corrects market failures which had been permitting firms to extract excess profits, reforms should move markets towards a competitive equilibrium, reducing margins while forcing firms to maintain competitive (lower) prices.^68^ This reality, that systematic consumer overpayment leads to systematic transfers of resources from consumers to the corporate sector,^69^ should be quite an obvious point in the aftermath of significant scandals such as the payment protection insurance (PPI) debacle—the regulatory response to which has restored approximately £50 billion in compensation to the real economy.^70^ Regulatory and academic studies increasingly demonstrate the magnitude of overcharging across the economy, and the potential billions of pounds which could be restored to consumers if the legal system withdrew its support for relevant pricing practices.^71^

## 3. Distributional Stakes of Legal Ground Rules: How Contractual Adjudication Can Distribute Resources

The idea that, from an aggregate perspective, (private) law shapes market prices and the distribution of resources among market participants should not be controversial. After all, it underpins probably the most-cited law review article of all time,^72^ Coase’s ‘The Problem of Social Cost’,^73^ which is much loved among conservatives and free-marketeers.^74^ Hale long ago taught us that ‘the market value of a property or service can be reduced to a combination of a party’s bargaining power under the particular legal rights with which the law endows him [or her]’.^75^ We now all accept that bargaining takes place ‘in the shadow of the law’, and that ‘legal rules create bargaining endowments’.^76^ Contemporary focus on inequality has highlighted how certain fundamental norms of our legal systems—including property law,^77^ corporate law,^78^ bankruptcy^79^ and competition law^80^—can establish ‘ground rules’ of capitalism which tend towards regressive distributions,^81^ while also often producing adverse macroeconomic effects.^82^ Arguments from the Great Moderation era of the 1990s that law should leave redistribution to tax-and-transfer systems^83^ now seem almost quaint after long periods of austerity and political, constitutional or market restrictions on public debt.^84^ No market exists without legal underpinning, and ‘[o]nce there is a legal system, the choice of any particular set of background rules is a choice of a set of distributive outcomes, whether achieved through many rules or only a few’.^85^ The law shapes market distributions both when it decides to leave bargaining solely to contract law norms and when a 500-page regulatory handbook prescribes detailed rules of conduct.

Certain perspectives on contracts underplay the law’s distributional role by framing contract law adjudication around the classic case of a freely negotiated agreement between equals, excluding cases outside these classic assumptions as matters to be addressed by legislation.^86^ This perspective ignores the crucial enduring role of contract law adjudication even in an age of widespread regulation. Legislation and regulatory rules will always struggle to keep pace with the arrival to market of new products, business models and pricing plans, leaving judges with the task of initial legal characterisation.^87^ Contract law is a first responder to urgent transformations such as the arrival of the ‘gig economy’, which has challenged existing legal categories and created new vulnerable classes who do not fit squarely within either consumer or employment legislation.^88^ In new markets for digital assets, crypto firms have sought to convince courts to withdraw cases from the scope of legislation by arguing that certain customers are not consumers, but rather ‘knowledgeable, experienced and sophisticated’ investors.^89^ Courts preside over the gaps which arise when regulation of one product type may cause firms to create ‘slightly different kinds of transactions that are functionally equivalent but outside the purview of the regulation’.^90^ The ‘buy now, pay later’ market, which advertises interest-free credit but imposes contingent charges on half of its borrowers, has seen its rapid growth take place outside the scope of current regulatory regimes.^91^ Finally, even for regulated products, it remains within courts’ interpretative power to determine the ultimate market impact of legislative and executive efforts—as clearly evidenced below in the UK Supreme Court’s application of unfair terms legislation. In this sense, ‘the judiciary have the first and last word’, shaping ‘ground rules’ and ‘legislative responses to these ground rules’.^92^ This suggests a need to recognise that ground rules are ‘made in large part by a group acting politically, but using a technique of rationalization that (a) denies the political component, and (b) justifies limited democratic control of their work’.^93^ In conditions of contemporary inequality, it becomes crucial to interrogate this rationalisation, and to examine the justifications for distributions produced by our courts.

This brings the discussion to the second sense in which the pricing practices and business models discussed in this article are contingent—their legality is far from inevitable.^94^ Indeed, their usage challenges key ideas of contract law. The foundational idea of ‘freedom of contract’ always contains a certain intellectual confusion,^95^ but neither of the two distinct modes of contractual reasoning supports the validity of contingent charges. Firstly, ‘broadly moral or philosophical’ justifications for upholding contracts,^96^ founded in ‘interpersonal’ rights and justice, ‘consent’ or a morality of contractual promising,^97^ generally do not extend to standard form contractual terms which have not been negotiated and do not in any real sense constitute ‘promises’. Secondly, ‘more scientific’ or instrumentalist approaches to contract,^98^ usually based on economic ‘policy’,^99^ cannot argue for the enforcement of contingent charging models in the face of empirical evidence of associated market failures. As a matter of contract law principle, the legal validity of firms’ demands for payment of contingent charges is an open question.

From a doctrinal perspective, when confronted with new markets and pricing practices, the common law gives considerable freedom to courts as to whether they should legitimate these business models. There is a ‘whole catalogue of common law approaches’ to problems arising in mass markets, with potential solutions based on such concepts as ‘fault’, ‘fair allocation of risk in light of business practices’ or the treatment of one-sided fine print ‘as if’ it represents agreed contractual terms.^100^ Statutory codifications of common law relating to sales contracts add quality standards and fitness-for-purpose guarantees, while even offering tortious strict liability for dangerous products.^101^ When legislators tasked the FCA with developing a price cap on ‘payday’ loans, the regulator considered most appropriate a total cost cap of 100% of the total amount borrowed.^102^ Intriguingly, this latter total cost cap resembles the *in duplum* rule existing under the common law of some jurisdictions.^103^ In short, contract law could readily regulate contingent charges through existing principles, most notably the penalty doctrine and unfair terms control. The UK Supreme Court had the opportunity to do so in the joined cases of *Cavendish Square Holding BV v Talal El Makdessi* and *ParkingEye Ltd v Beavis*.^104^

## 4. Presenting the Facts while Moving Beyond Microjustice and Bilateral Models of Adjudication

The above discussion illustrates how contract law adjudication serves dual functions—it *distributes resources*, just as much as it *resolves individual disputes* ‘in ways that people find more or less fair or just’.^105^ These dual functions are illustrated by the notable differences between the cases joined before the Supreme Court. The *Makdessi* case exemplifies the latter form of contractual adjudication. It was a multi-million-dollar case involving a bespoke deal to sell a majority stake in ‘the largest advertising and marketing communications group in the middle East’. Here, ‘both sides were represented by highly experienced and respected commercial lawyers’.^106^ Under the terms of the contract, the sellers were not to carry out activities which competed with those of the purchasers, and clauses provided that the sellers would lose entitlement to certain payments and shareholdings on breaching these obligations. The Supreme Court upheld the validity of these clauses and declined to categorise them as unenforceable ‘penalty clauses’.

In contrast, the *ParkingEye* case lies at the other end of a dispute–distribute scale, establishing rules governing an entire market. It involved a consumer’s use of a car park, which the operator, ParkingEye, ran according to a model of contingent charging. ParkingEye offered free time-limited parking in the relevant car park, in which it had displayed a number of signs stating

‘2 hour max stay’,

‘Parking limited to 2 hours’, and

‘Failure to comply with the following will result in a Parking Charge of £85’.

Under the agreed facts, Mr Beavis parked in the car park for a period of two hours 56 minutes. ParkingEye sent letters to the consumer’s address, demanding first £50 (on prompt payment)^107^ and then £85.^108^ Mr Beavis refused to recognise these demands as legal. He challenged the relevant parking charges under both the common law penalty clause doctrine and the protection from unfair terms under the Unfair Terms in Consumer Contracts Regulations 1999.^109^ The unfair terms legislation includes an indicative list of terms which may be unfair, among which is listed a term which requires a consumer to pay ‘a disproportionately high sum in compensation’ on breach of contract.^110^

Mr Beavis’s crowd-funded appeal was not a dispute between two parties over a sum of £85.^111^ Rather, in this test case, the Supreme Court was asked to lay down ground rules which would apply across the market for car park services. The overall size of this market is difficult to determine, but a recent House of Lords Library report referred to a ‘billion-pound’ market and cited an estimate that private parking operators issued consumers with £2.62bn of parking and traffic charges in the year 2021–22, an increase from £1.63bn in 2017–18.^112^ ParkingEye describes itself as ‘the largest private sector operator of ANPR [Automatic Number Plate Recognition] car park management’.^113^ It was valued at £235m in a 2018 purchase by global asset management giant Macquarie, apparently the owner of infrastructure on which over 100 million people rely daily.^114^ A freedom of information request by Professor Iain Ramsay has found that ParkingEye Ltd is the ninth highest issuer of County Court proceedings in the country, with 33,171 bulk claims in the County Court Business Centre in 2019–20.^115^ It thus plays a large part in the practice of ‘assembly-line’ litigation,^116^ and the routinised court processing of corporate debt collection.^117^ In a recent speech advocating for alternative dispute resolution policies which would continue trends of removing ‘small’ matters from the courts, Sir Geoffrey Vos, Master of the Rolls, commented that it would be ‘obviously absurd’ if any jurisdiction allowed parties to challenge parking tickets before the Supreme Court.^118^ If we view a parking ticket matter as involving a dispute between two parties over a bill of £85, it may indeed seem absurd.^119^ It is less so if we view the matter as involving a question of whether hundreds of millions of pounds per year should be distributed to consumers or parking firms.^120^

This joint opinion is most commonly referred to only as the *Makdessi* case,^121^ perhaps revealing a general tendency of contract lawyers, and arguably judges, to focus reasoning on the ‘classic case’ of freely negotiated bespoke business-to-business deals.^122^ The paradox of this approach is that we have known for decades that formal contracts are less significant in business relationships.^123^ It is in firms’ claims against consumers that contracts are enforced routinely and to their letter.^124^ Focus on the paradigm of business-to-business negotiation thus risks developing contract law based on less representative scenarios, ignoring how our economy is shaped by unread standard form documents.

A view of contract law adjudication as a bilateral matter of ‘interpersonal justice’^125^ also tends towards the exclusion of distributive questions. Prevailing arguments against distributive considerations in contract law, in both philosophical and economic approaches,^126^ focus on private law adjudication as an isolated matter between two litigants. From this position, these accounts often reason that contract law should not compensate for external and systemic regressive distributions, by favouring a party who may be considered a victim of some societal injustice unrelated to a particular inter-party dispute. This approach does not contemplate that contract law rules and adjudications may themselves *create* regressive distributions.

Conversations framed around hypothetical disputes between abstract parties, with random income differentials caused by unidentified external factors,^127^ may conceal evidence of extant material inequality and class conflict.^128^ They also offer few suggestions as to how to decide a case like *ParkingEye*, in which a court is asked directly to distribute, by upholding or denying the claims of an industry to transfer resources to itself from consumers. A case-by-case view of the law risks a ‘preoccupation with microjustice’, and a failure to see the ‘broad consequences of law and legal institutions’.^129^ If we only look at cases of bilateral dispute resolution like *Makdessi*, we will miss the type of market regulation in which the Supreme Court was forced to engage in *ParkingEye*.

## 5. The ‘Price’ Paradox: When a Court Decides That More Revenue Means Less Regulation

A significant aspect of the indeterminacy of contract law, and the contingent legality of any pricing or product design, is the initial question as to where to situate a practice among various possible legal categories. A key principle of contract law dictates that, even while policing penalty clauses and other unfair terms, a court should not interfere with terms which are categorised as forming the ‘primary obligations’ or ‘core bargain’ struck by the parties—most importantly, the price. The boundaries of this excluded zone will determine the freedom of firms to design contingent pricing models which not only evade competitive market discipline, but also escape scrutiny under contract law. The penalty rule has traditionally applied only to contractual terms which are triggered by a party’s breach of contract. The Supreme Court in *ParkingEye* effectively upheld this position, stating that the only clauses covered by the doctrine are ‘secondary obligations’ which create contractual alternatives to court-determined damages for breach of contract.^130^ If the impugned clause is merely a ‘conditional primary obligation’, then the court will have no jurisdiction to evaluate the clause as a potential penalty. The distinction between secondary obligations and ‘conditional primary obligations’ has been criticised as being so vague as to be unworkable,^131^ but this may be quite deliberate. The Court expressly acknowledged that this new test invites firms to draft contracts to avoid the penalty doctrine.^132^ It dismissed the old law’s safeguards against lawyers converting alleged penalties into immune ‘price’ clauses.^133^ Therefore, while formally the penalty doctrine remained intact, the Supreme Court significantly narrowed its scope of application.

This hands-off approach to the control of penalties bears remarkable similarities to the Supreme Court’s previous expansion of the key exclusion in unfair terms legislation—the exemption from fairness review of ‘the main subject matter of the contract’ or ‘the appropriateness of the price payable under the contract’.^134^ In *Office of Fair Trading v Abbey National*, following the arguments of counsel Jonathan Sumption QC for the banking industry, the Court rejected the view of a specialist regulator that unarranged overdraft fees fell outside of this price exemption, and so should be subject to fairness review.^135^ The sparse reasoning of the Supreme Court seemed to rely heavily on the fact that banks derived over 30% of their revenues from unarranged overdraft charges, and that such a significant source of revenue must be part of the ‘price’ of bank account services.^136^ This reasoning introduces a ‘too big to fail’ imperative to contract law’s regulation of pricing structures, producing the surprising outcome that the more revenue an industry manages to extract from consumers through contingent pricing, the less likely will it be that the law will intervene.^137^

The Supreme Court’s reasoning across both cases validates contingent pricing practices, allowing firms to offer low headline prices while raising revenues through shrouded costs holding formal legal categorisation as untouchable ‘prices’ or ‘(conditional) primary obligations’. Counsel in the *Makdessi* and *ParkingEye* hearings had argued for an alternative approach, following the High Court of Australia, which would apply the penalty doctrine to all onerous terms which were conditional on a failure to observe another term, irrespective of technical definitions of contractual breach.^138^ The Australian court’s approach was designed to prevent parties from drafting around the penalty doctrine. It emanated from a perspective which recognised the existence of significant ‘remedial legislation’ in relevant banking markets, and took this as a reason to be sceptical of the idea that ‘*laissez faire* notions of an untrammelled “freedom of contract” provide a universal legal value’.^139^ In rejecting this perceived expansion of the penalty doctrine, the UK Supreme Court in *ParkingEye* took the ‘growing importance of statutory regulation in this field’ as suggesting the opposite conclusion—that courts should lean further towards a *laissez faire* contract law. Its limitation of judicial power to control penalties followed a general condemnation of the doctrine as ‘an inroad upon freedom of contract’ and an anxiety not to expand ‘the courts’ supervisory jurisdiction into a new territory of uncertain boundaries, which has hitherto been treated as wholly governed by mutual agreement’.^140^

The judges presented a puzzling understanding of ‘mutual agreement’.^141^ Most philosophical defences of contractual freedom exclude from their study unread standard form contracts. Economic defences of boilerplate similarly rely on the idea of hypothetical consumer consent, rather than pretending that such terms are scrutinised by consumers.^142^ It is jarring, therefore, to see Lords Neuberger and Sumption apparently placing weight on their view that motorists, and Mr Beavis, in fact ‘did accept’ the terms of ParkingEye’s contract.^143^ When they subsequently considered the hypothetical question of whether a consumer *would have* accepted the standard terms offered by ParkingEye,^144^ it became clear that the consumer the judges had in mind was a rational actor who benefited from free access to a car park which is not ‘clogged up with commuters and other long-stay users’.^145^ The Court did not seem to contemplate that a ‘long-stay user’, including one who inadvertently remains parked for more than 59 minutes, might also fall into the category of hypothetical consumers.

The Court of Appeal in *Abbey National* demonstrated an alternative approach of assessing hypothetical consumer agreement, while offering a more enlightened solution to the ‘price’ puzzle. In so doing, it showed a path to using unfair terms legislation to police contingent charges, in a manner more compatible with contemporary regulatory thinking. The judgment of Sir Anthony Clarke MR considered that unarranged overdraft fees fell outside the definition of ‘price’ for the reason that consumers are unlikely to take into account these contingent charges when choosing a bank account.^146^ It accepted Office of Fair Trading arguments that the price exclusion aimed to remove from judicial control ‘that part of the bargain that will be the focus of a customer’s attention when entering into a contract’, since ‘market forces could and should be relied upon’ to control these visible terms.^147^ This analysis reflects academic explanations of unfair terms legislation as implementing the ‘market for lemons’ model of information asymmetries.^148^ It also accords with contemporary understandings of how contingent pricing strategies are designed to exploit consumer error to evade competitive pressures.^149^ The UK Supreme Court rejected this reasoning as irrelevant to the issue of whether a charge fell within the ‘price’. In an apparent *non sequitur*, considering the reasoning’s clear foundation in market failure analysis, Lord Phillips dismissed this argument as raising non-justiciable questions of ‘whether the method of pricing is fair’ and the general equity of cross-subsidisation.^150^ While proponents of the Court of Appeal’s argument may well believe that the poor should not pay more, on this occasion the reasoning seems to confront directly the logic of efficiency through market failure analysis, rather than challenging the court to a tangential equity–efficiency debate.^151^

## 6. Institutional Competence: Should Courts Judge the ‘Legitimacy’ of Business Models?

An ‘important legal system policy is that a legal agency should hesitate before it attempts a task it is unlikely to do very well’.^152^ A key reason why certain commentators feel content that contract law can ignore questions of distribution and perhaps even market efficiency is founded in a typical argument from institutional competence^153^—the idea that these considerations are most effectively left to legislators (suited to taking ‘political’ decisions) and expert regulators (who can conduct empirical analysis beyond the abilities of a court).^154^ Indeed, the *Abbey National* decision begins with an apologia, in which Lord Walker insists that the court’s ‘limited’ role is not to decide on the fairness of the ‘reverse Robin Hood exercise’ represented by the operation of the unarranged overdraft market.^155^ In classic depoliticising language,^156^ the judge emphasised that the court was deciding a mere ‘technical’ question. One might imagine that such an understanding of courts’ appropriate role would see judges adhere closely to doctrinal precedent and limit the judicial role within traditional boundaries. Courts who express relief that ‘fortunately’ policy questions are ‘for Parliament and not for this court’^157^ might wish to defer heavily to legislative authority and regulatory expertise. Contrary to these expectations, in relation to the question of the appropriate standard for assessing the validity of default charges which cannot be squeezed into the spacious ‘conditional primary obligation’ or ‘price’ categories, the Court in *ParkingEye* seemed to stray towards a more activist style.

Historically, the penalty doctrine permitted contracts to stipulate default charges payable on breach (under ‘liquidated damages’ clauses), so long as the specified amount did not exceed a genuine pre-estimate of the loss caused by the breach.^158^ The Supreme Court in *ParkingEye* reinterpreted this position to such a degree as to overrule precedent in substance, if not in form.^159^ Lords Neuberger and Sumption reformulated the relevant standard in stating that the ‘true test is whether the impugned provision is a secondary obligation which imposes a detriment on the contract-breaker out of all proportion to any legitimate interest of the innocent party in the enforcement of the primary obligation’.^160^

This represented a relaxation of the standard of the penalty test, allowing clauses to be valid even where they exceeded reasonable predictions of losses associated with contractual breach. Alongside this permissive approach to the penalties test, the second deregulatory step taken by Lords Neuberger and Sumption, which provoked a dissent from Lord Toulson, was to collapse the standard for statutory unfair terms review into this newly relaxed common law paradigm. The relevant legislative test for unfairness asks whether a contractual term, ‘contrary to the requirement of good faith’, ‘causes a significant imbalance in the parties’ rights and obligations under the contract to the detriment of the consumer’. Despite the apparent aim of the legislation to correct market failures and to expand regulation of contract terms beyond traditional common law rules, Lords Neuberger and Sumption took the view that ‘the same considerations which show that the … charge is not a penalty, demonstrate that it is not unfair for the purpose of the [legislation]’.^161^ The judges found that the relevant charge was not contrary to the requirement of good faith, since ParkingEye had a ‘legitimate interest’ in levying it on customers. In so doing, the judges brushed past, without explanation, the structure of the unfair terms legislation, which establishes that default/penalty charges are presumptively invalid.^162^

Under the old standard of the penalty doctrine, ParkingEye’s £85 charge would clearly have constituted an invalid penalty, since the firm suffered no loss from a customer parking for more than two hours.^163^ The application of this traditional test confined judges to very familiar territory, only requiring them to carry out the classic judicial task of resolving a dispute as to the losses caused to one party by the other’s contractual breach.^164^ The new approach, of judging the ‘legitimacy’ of a firm’s interest in compelling performance, expands the judicial role significantly.^165^ It seizes territory more usually held by legislators and regulators in asking judges to decide whether a pricing model might be in the best interests of ‘the general body of users’ or ‘the public at large’. Lords Neuberger and Sumption held that ParkingEye had a legitimate interest in charging customers ‘which extended beyond the recovery of any loss’. They stated that the interest of ParkingEye was in controlling access to the car park and imposing the relevant charges ‘with a view to managing the car park in the interests of the retail outlet, their customers and the public at large’.^166^ The judges identified the ‘perfectly reasonable’ objectives of the charge as being both ‘to manage the efficient use of parking space in the interests of the retail outlets, and of the users of those outlets’, and ‘to provide an income stream to enable ParkingEye to meet the costs of operating the scheme and make a profit from its services’. They took the view that ‘the imposition of a charge to deter overstayers is a reasonable mode of achieving these aims’,^167^ and that ‘charging overstayers £85 underpinned a business model which enabled members of the public to park free of charge for two hours’.^168^

This reasoning appears to amount to a general endorsement of the legitimacy of the business model currently operated by ParkingEye. It is an implicit statement that this must be an efficient, or even the most efficient, manner of operating a car park. However, even if ‘it is difficult [for the Court] to see’ alternative business models,^169^ it surely is not beyond the ingenuity of firms to develop competing pricing structures, or outside the abilities of regulators to iron out identifiable inefficiencies. Rather than affirming the efficiency of the contingent pricing model, detailed market analyses could potentially point to a range of possibly more efficient equilibria. A court which disavows its traditional role as a mere adjudicator of losses to pronounce on the legitimacy of business models and their ability to benefit the ‘public at large’ would need to show that it can conduct the market analysis necessary to support claims that a business model serves the public interest.

The Supreme Court’s reasoning in *ParkingEye* illustrates that it has no appetite for engaging in such analysis. While the shift to the new ‘legitimate interest’ test resembles a statement of policy change both in language and in its contingency,^170^ Lords Neuberger and Sumption professed to follow the traditional private law process of ‘discovering’ the new ‘true’ test in old precedents.^171^ The ‘legitimate interest’ test was found in dubious beginnings, however, as Lords Neuberger and Sumption linked it to the historical *Dunlop* case.^172^ Here, tyre manufacturer Dunlop included a resale price maintenance clause in its standard form supply contracts with garages, breach of which carried a specified charge. Lords Neuberger and Sumption considered that this contractual structure was based on Dunlop’s legitimate interest in protecting the manufacturer’s brand and market position, which could be undermined if garages had freedom to sell Dunlop products at a lower, more competitive price. This reasoning is remarkable, given that resale price maintenance, or vertical price fixing, is an illegal ‘hardcore’ restriction of competition.^173^ The Supreme Court thus drew the ‘legitimate interest’ test from an apparent idea that a price-fixing practice in breach of basic rules of market regulation would be a ‘legitimate’ business interest. As well as suggesting a hostility to the idea that contract law could be shaped by even the most fundamental ideas of contemporary regulatory analysis, this approach makes it very difficult to see what current market practice the Supreme Court might ever consider *illegitimate*. The logical conclusion of this reasoning perhaps arrived in the Australian decision of *Paciocco*, where the High Court accepted that the mere profitability of a firm can be a legitimate interest.^174^ Will contract law now consider any practice of a company which produces revenue, whether legal or in violation of basic regulatory rules, as serving a legitimate interest?

The Supreme Court’s conviction in the efficiency of current business practices stands in stark contrast to its judgment of consumer behaviour. The car park was assumed to be operating efficiently and in the public interest. In sharp contrast, the Court held consumers to a hypothetical standard of rational efficiency, irrespective of the extant market reality of consumer behaviour. Mr Beavis was expected to leave the car park on time, having rationally weighed the costs and benefits of the parking decision—no allowances were made for time-inconsistent preferences, optimism biases or even the reality that car park signs may not have been read. Lord Mance rejected arguments founded in behavioural economics that the ‘scheme only works by taking advantage of human fallibility or unforeseen circumstances’. He denied that it ‘relies on human (over)optimism’ that no circumstances will arise to cause an overstay.^175^ When lawyers provided precedent of the High Court interpreting unfair terms legislation in light of the concept of consumer optimism bias,^176^ Lord Mance rejected this decision as being too ‘fact sensitive’^177^ to offer wider authority beyond its context of specific gym membership contracts.^178^ Similarly, Lords Neuberger and Sumption outright refused to accept evidence of the Consumers’ Association that motorists may overstay due to unforeseen circumstances.^179^ According to the judges, the ‘risk of having to pay … was wholly under the motorist’s own control. All that he needed was a watch.’^180^

This asymmetrical reasoning presumes the efficiency of business practices while condemning the actual consumer behaviour of those who incur charges as ‘infractions’ or a failure to ‘observe the rules’.^181^ Parallels can be seen in Lord Walker’s search for blame in overdraft markets in *Abbey National*. He describes the fairness of unarranged overdraft charges as ‘an imponderable question which depends partly on whether one’s perception of the average customer who incurs unauthorised overdraft charges is that he is spendthrift and improvident, or that she is disadvantaged and finding it hard to make ends meet’.^182^

Models grounded in realities of consumer behaviour rather than abstract concepts of rationality—tinged with neoliberal judgment of irrational economic actors^183^—would render this question less imponderable and more empirical. In contrast, an unwillingness to accept without condemnation the reality of systematic consumer error, while assuming without question the efficient rationality of firms, inevitably leans the law heavily towards upholding business claims, irrespective of consumer harm. If a motorist needs only a watch, would the Supreme Court also say that problems in markets for 1000% APR payday loans or overdrafts could be solved by supplying households with calendars?

## 7. Endorsing Misaligned Incentives and Ending Mutual Benefit in Contracts

Both moral and economic perspectives share an idea that mutual benefit lies at the heart of contract. According to legal orthodoxy, ‘it remains universally the case that parties to a proposed contract bargain as to its terms so that each side will benefit’,^184^ mirroring the ‘economists’ ideal where both leave the bargaining table in a better position than when the negotiations began’.^185^ Contingent charges strike at the heart of this conception of contract, by misaligning the incentives of consumer and firm. Rather than both firm and consumer interests being maximised by the performance of the contract as originally envisaged, a firm’s interests may be best advanced by an unanticipated or underappreciated outcome detrimental to the consumer.

The Supreme Court’s stated test for penalty clauses involves the question of whether a firm has a legitimate interest *in the performance of the primary obligation*. The Court clarified that an interest in deterring breach of contract could be regarded as legitimate, in a departure from prior law.^186^ It emphasised that ParkingEye’s charge served the object of rationing ‘scarce parking space’ and of ‘simply influenc[ing] the behaviour of motorists by causing them to leave within two hours’.^187^ Lords Neuberger and Sumption took support from statements of Advocate General Kokott of the Court of Justice of the European Union, who had similarly explained that a default interest charge in a credit contract might be justified where it ‘motivates the debtor not to default’.^188^ This reasoning shows a certain naivety as to the revenues to be generated from contingent charges and their centrality to contemporary business models.^189^ After all, if we think firms are only concerned with promoting performance in this way, they would be equally satisfied with consumers, say, donating money to charity on default.^190^

More directly, the obvious criticism of this reasoning lies in Lord Neuberger and Lord Sumption’s admission that ParkingEye’s ‘revenues are wholly derived from the charges for breach of the terms’.^191^ This means that if the £85 default charge was successful in advancing ParkingEye’s supposed interest in encouraging every motorist to perform their contracts and leave car parks within two hours, the car park operator would presumably cease to exist as a profitable venture. The reality is that it is in the car park operator’s interest that a significant number of consumers default. In perhaps even starker terms than the high-cost lender or bank which profits from consumers’ inability to repay loans or honour transactions, the car park operator was entirely dependent for revenue on consumers ‘breaching’ the original contractual terms. The ‘innocent’ firm would not survive profitably unless it was confident that it could lure sufficiently high numbers of ‘contract-breakers’, who would fail to ‘observe the rules’ and so become liable to unexpected demands for default charges.^192^ The traditional penalty doctrine’s approach of tying contractual damages to losses caused by breach had the merit of removing incentives for contract drafters to lay ‘traps’ for their counterparts,^193^ ensuring that it would not be more profitable for a drafting party to see its counterpart default rather than perform the contract. As commentators and the Supreme Court search for rationales behind the penalty clause prohibition,^194^ a compelling answer seems to derive from the imperative of preserving the centrality of mutual benefit to contract law.

## 8. Relativist Assessment of Pricing in an Equilibrium of Manipulation

In assessing the compliance of the *level* of the default charge both with the penalty rule and unfair terms legislation, the Supreme Court took the view that the ‘amount of the charge was not exorbitant in comparison to the general level of penalties imposed for parking infractions’.^195^ In particular, Lords Neuberger and Sumption agreed with the trial court that ‘the £85 charge was neither extravagant nor unconscionable having regard to the level of charges imposed by local authorities for overstaying in car parks on public land’.^196^ For the judges, this cross-sector comparison spoke both to the fairness of the charge and to its proportionality to ParkingEye’s legitimate interest in its customers’ contractual performance. This shows a relativist approach to judging charges, which for its efficacy will depend on a high level of market competition and an absence of sector-wide market failures. This is problematic in the parking sector, as shown immediately by the courts’ perhaps misplaced reliance on local authorities as benchmarks of reasonableness. In the face of austerity and severe cuts in central government funding, local authorities have become increasingly commercialised, operating largely as (often monopolistic) market actors.^197^ To a growing degree, they rely on raising revenue through tools such as parking fines.^198^ Council parking fees increased by over 40% in the years from 2013–14 to 2018–19, reaching a high of £1.7 billion in revenue (£934 million in profit).^199^ Councils have also developed a reputation for aggressive debt collection techniques, with a House of Commons committee branding local authorities as ‘the most zealous and unsympathetic of creditors’.^200^ Local authorities in England and Wales referred parking charges to bailiffs in over one million cases in 2018–19.^201^ Scrutiny of local governments’ regressive recourse to mechanisms such as parking fines for revenue has, in turn, drawn attention to disproportionate impacts of parking fines on low-income localities.^202^

Looking more broadly beyond this specific sector, an approach of comparing prices charged across a market misses the point that contingent prices prevail in uncompetitive conditions where prices are shielded from market discipline. Lords Neuberger and Sumption suggested that none of their analysis ‘means that ParkingEye could charge overstayers whatever it liked’. The very problem of contingent pricing is that it evades competitive scrutiny by exploiting behavioural biases and information asymmetries—meaning that whole industries of firms can effectively charge whatever they like. This is the manipulative equilibrium identified by Akerlof and Shiller—once one firm has figured out a means of playing on consumer biases to increase revenues, competitive imperatives mean that rival firms must match their pricing practices.^203^ In *Paciocco*, Keane J of the Australian High Court (perhaps unintentionally) admitted this limitation of relativist analysis in suggesting that it would be ‘something of a stretch’ to sanction as unfair the conduct of one market participant engaging in commonplace activities without arguing that the entire market is ‘unlawfully skewed’.^204^ The problem is that, as the examples above show, regulators frequently find examples of such ‘skewed’ markets. Where unarranged overdraft interest rates regularly exceed 10% per day, a daily charge of 9% or 11% could hardly be considered disproportionate on a relativist scale. But few could dispute the FCA’s view that these ‘charges are high in an absolute sense’.^205^ A relativist approach to comparing market rates may be suited to a bilateral model of adjudication, where an interpersonal claim is based on the argument that one contracting party has committed a singular wrong against its counterpart. It offers no chance of a legal remedy in a test case arising from an uncompetitive market equilibrium of systematic consumer error. Rather than engaging in empty assessments of the proportionality or fairness of charges based on prevailing (uncompetitive) market conditions, it may have been more appropriate for the courts to have preserved a traditional judicial role of measuring actual losses caused by contractual breaches.^206^

## 9. The Law and Political Economy of Contract: Business as Usual in the Supreme Court?

The response to the *ParkingEye* decision from Civil Enforcement Ltd, a major parking charge processor and issuer of County Court claims, is to greet all visitors to its website (usually directed there by demands for payment) with the message ‘Is my Parking Ticket Enforceable? On 4th November 2015 the UK Supreme Court removed any doubts over the legality and enforceability of Parking Charge Notices issued for parking on private land.’^207^

This triumphant position clearly illustrates the parking industry’s view that the Supreme Court had legitimated its business model and enhanced the credibility of its debt collection letters.^208^ Apparently the *ParkingEye* decision now appears routinely, via ‘cut and paste’ references, in parking charge claims under which county courts are currently ‘deluged’.^209^ Seemingly in response, a Conservative government not known for its regulatory zeal made multiple efforts to introduce statutory regulation. In the face of industry opposition, including successful legal challenges to plans for a proposed Code of Practice, the government has revisited its proposals.^210^

This story reminds us of the challenges of legislative and regulatory reform,^211^ particularly in a market in which incumbents have grown strong through amassing fees under weak competitive conditions. The unarranged overdraft story offers further evidence on this point, as the *Abbey National* case itself represented banking industry mobilisation before the Supreme Court to stop the relevant expert regulator from using consumer protection legislation to police these fees.^212^ It was almost 10 years later that the FCA published regulations to eliminate the more egregious practices in this sector.^213^ One must wonder at the distribution of resources from consumers to the financial sector in this billion-pound market during the intervening decade. Those who would dismiss from court adjudication any questions of distribution, macroeconomic impact or even more basic market efficiency must accept the reality that a court faced with a failing market may have ‘the last word because the doors to the legislature are closed as a result of the balance of economic and political power in the situation’.^214^ Judges might not always be able to assuage their consciences through confidence that any social or economic injustice resulting from their ‘technical’ decisions will be readily alleviated by prompt political action.

Contract law cannot save us. This article does not present an agenda for the overhaul of private law, or even contract law, to serve a singular aim of redistribution or social justice.^215^ On the other hand, contract law can certainly harm us, and this article urges acknowledgement of the reality that, in some cases, contractual adjudication has market-shaping and distributive consequences. In these situations, despite the impression of technicality, ultimately courts can be seen to be allocating resources in a manner we might recognise as *political*, as much as legal. Particularly where these allocations are regressive, it becomes essential to interrogate their underpinning justifications.^216^ When Griffith broke the courtroom’s fourth wall half a century ago, he revealed that judges ‘cannot avoid the making of political decisions. What is important is to know the bases on which such decisions are made.’^217^ If sufficient justifications are not offered, whether founded in accepted moral or economic understandings of contractual reasoning, courts who uphold firms’ demands for payment risk being seen as merely protecting incumbent market actors, ‘supporting the existing allocation of power in society’.^218^

In the early days of the neoliberal turn, Atiyah linked legal and political philosophy in suggesting that the ‘message of the New Right [was] being heard in the law courts as well as in the City of London’.^219^ For Atiyah, this trend re-established ‘Freedom of Contract … as the ideology of the common law’.^220^ This is a rare recognition of the political stakes in English contract law adjudication. While academic commentary and public debate frequently consider the extent to which decisions of the US Supreme Court can be categorised as ‘pro-business’,^221^ the UK Supreme Court seems to be spared similar scrutiny.^222^ The distributional effects of private law often remain shrouded ‘by an ideological view of private law as a neutral set of ground rules which are rationally developed by the judiciary on the basis of principle and precedent’.^223^ Certain judges, practitioners and academics persist in viewing this area of judge-made law as ‘the antithesis of politics’, a technical doctrinal field of reason that can be contrasted with a public law sphere of political *will* manifested in legislation, regulation and related norms.^224^

Yet it becomes difficult to read decisions like *ParkingEye* and not detect ideological leanings of the type identified by Atiyah. Neoliberalism is often associated with the diffusion of economic values and frameworks to ‘spheres and activities heretofore governed by other tables of value’.^225^ One prominent criticism, however, is that the use of economic ideas under neoliberal policymaking is instrumental and selective,^226^ or of an overly simplistic and fundamentalist nature.^227^ Alongside the ‘market neoliberals’ who support regulation where market failure analysis requires it, have emerged the pro-business ‘corporate neoliberals’—who defend the role of incumbent firms even if the result is to ignore market failures,^228^ and trade authentic economic analysis for a faith of market fundamentalism.^229^

It is hard not to see some of these trends emerging in Supreme Court reasoning, which declares, in the face of widespread evidence of corporate exploitation of systematic consumer error, that a contingent charging practice will be less likely to be reviewable the more income it produces for incumbent firms. One must raise similar questions when a court reshapes common law and ‘waters down’^230^ statute to declare itself arbiter of the legitimacy of business models, before establishing criteria under which any revenue-raising practices will be legitimate and the only test of legality of charges is the prevailing (potentially uncompetitive) market rate. These approaches may well work in bilateral disputes between equally rich parties relating to one-off deals they have freely negotiated. This article has aimed to show the emptiness of this reasoning when applied to complex pricing models such as contingent charging, which are crafted into standard form contracts and deployed on mass markets to evade competition and reap supra-normal profits. If ‘freedom of contract’ means enforcing incumbent firms’ demands for payments under such conditions, in circumstances where enforcement is justified neither by moral nor by economic strands of contractual reasoning, one must wonder whether this concept operates as a meaningful legal principle or mere ‘conservative policy argument’.^231^ As we face a cost-of-living crisis which threatens to depress living standards and deepen inequality, it may be no longer possible to ignore the distributional effects and inherent politics of contract law adjudication. Can we afford to continue with business as usual?

## Footnotes

1 Joseph Stiglitz, *The Price of Inequality: The Avoidable Causes and Invisible Costs of Inequality* (Allen Lane 2012) ix.

2 Piketty offers perhaps the most widely noted version of this critique: Thomas Piketty, *Capital in the Twenty-First Century* (Harvard UP 2014); Heather Boushey, J Bradford Delong and Marshall Steinbaum, *After Piketty: The Agenda for Economics and Inequality* (Harvard UP 2017).

3 Barack Obama, ‘Remarks by the President on Economic Mobility’ (The Arc, Washington DC, 4 December 2013) <https://obamawhitehouse.archives.gov/node/248126> accessed 14 June 2018.

4 The groups with the highest ‘marginal propensity to consume’: Atif Mian and Amir Sufi, *House of Debt* (University of Chicago Press 2014).

5 On macroeconomic risks of inequality: Anthony B Atkinson, *Inequality* (Harvard UP 2015) 243–62; Era Dabla-Norris and others, ‘Causes and Consequences of Income Inequality: A Global Perspective’ (IMF 2015) IMF Staff Discussion Note.

6 William Davies and others, *Unprecedented?: How COVID-19 Revealed the Politics of Our Economy* (Goldsmiths Press 2022) 75.

7 Josh Bivens, ‘Corporate Profits Have Contributed Disproportionately to Inflation. How Should Policymakers Respond?’ (Economic Policy Institute 2022); Isabella Weber and Evan Wasner, ‘Sellers’ Inflation, Profits and Conflict: Why Can Large Firms Hike Prices in an Emergency?’ I2023O Economics Department Working Paper Series <https://scholarworks.umass.edu/econ_workingpaper/343>.

8 Piketty (n 2).

9 Susanne Soederberg, *Debtfare States and the Poverty Industry: Money, Discipline and the Surplus Population* (Routledge 2014) 37; David Harvey, *The Enigma of Capital: And the Crises of Capitalism* (Profile Books 2011) 66–8; ‘when we are underpaid relative to the value of what we produce, we experience labour exploitation. And when we are overcharged relative to the value of something we purchase, we experience consumer exploitation’: Matthew Desmond, *Poverty, by America* (Crown Publishing Group 2023) 63.

10 Stiglitz (n 1).

11 Oren Bar-Gill, *Seduction by Contract: Law, Economics, and Psychology in Consumer Markets* (OUP 2012) 2. Emphasis in the original.

12 George A Akerlof and Robert J Shiller, *Phishing for Phools: The Economics of Manipulation and Deception* (Princeton UP 2015) vii.

13 Federal Trade Commission, Unfair or Deceptive Fees Trade Regulation Rule, Commission Matter No R207011, Advance notice of proposed rulemaking; request for public comment; The White House, ‘The President’s Initiative on Junk Fees and Related Pricing Practices’ (2022) <www.whitehouse.gov/briefing-room/blog/2022/10/26/the-presidents-initiative-on-junk-fees-and-related-pricing-practices/> accessed 13 February 2023.

14 Mark Armstrong and John Vickers, ‘Consumer Protection and Contingent Charges’ (2012) 50 Journal of Economic Literature 477.

15
*Cavendish Square Holding BV v Talal El Makdessi; ParkingEye Ltd v Beavis* [2015] UKSC 67.

16
*The Office of Fair Trading v Abbey National plc & Others* [2010] 1 AC.

17 This was Lord Toulson’s description of the approach of the majority towards unfair terms legislation in ParkingEye: *ParkingEye* (n 15) para 315.

18 Duncan Kennedy, ‘The Political Stakes in “Merely Technical” Issues of Contract Law’ (2002) 10 European Review of Private Law7, 7.

19 Desmond (n 9) ch 4.

20 The White House, ‘Remarks of President Joe Biden—State of the Union Address as Prepared for Delivery’ (2023) <www.whitehouse.gov/briefing-room/speeches-remarks/2023/02/07/remarks-of-president-joe-biden-state-of-the-union-address-as-prepared-for-delivery/> accessed 13 February 2023.

21 Kennedy, ‘Merely Technical’ (n 18) 7.

22 Tony Prosser, ‘Regulation and Social Solidarity’ (2006) 33 JLS 364, 366–71.

23 Luke Herrine, ‘What Is Consumer Protection For?’ (2022) 34 Loyola Consumer Law Review.

24 See eg Iain Ramsay, *Consumer Law and Policy: Text and Materials on Regulating Consumer Markets* (3rd rev edn, Hart Publishing 2012) 41–54.

25 Akerlof and Shiller (n 12) x.

26 Vijay Raghavan, ‘Consumer Law’s Equity Gap’ [2022] Utah L Rev 26.

27 Bar-Gill, *Seduction by Contract* (n 11) 23–5.

28 Xavier Gabaix and David Laibson, ‘Shrouded Attributes, Consumer Myopia, and Information Suppression in Competitive Markets’ (2006) 121 Quarterly Journal of Economics 505.

29 George A Akerlof, ‘The Market for “Lemons”: Quality Uncertainty and the Market Mechanism’ (1970) 84 Quarterly Journal of Economics 488.

30 Yannis Bakos, Florencia Marotta-Wurgler and David R Trossen, ‘Does Anyone Read the Fine Print? Consumer Attention to Standard-Form Contracts’ (2014) 43 JLS 1.

31 Oren Bar-Gill, ‘The Law, Economics and Psychology of Subprime Mortgage Contracts’ (2008) 94 Cornell L Rev 1073, 1096–100.

32 Iain Ramsay, ‘From Truth in Lending to Responsible Lending’ in Andre Janssen and Geraint Howells (eds), *Information Rights and Obligations: The Impact on Party Autonomy and Contractual Fairness* (Avebury Technical 2005) 53; Ron Harris and Einat Albin, ‘Bankruptcy Policy in Light of Manipulation in Credit Advertising’ (2006) 7 Theoretical Inquiries in Law 431, 434.

33 Joseph Spooner, *Bankruptcy: The Case for Relief in an Economy of Debt* (Cambridge UP 2019) 58–9.

34 Bar-Gill, ‘The Law, Economics and Psychology’ (n 31); Financial Conduct Authority (FCA), ‘From Advert to Action: Behavioural Insights into the Advertising of Financial Products’ (2017).

35 Elizabeth Warren, ‘Balance of Knowledge’ in Ralph Brubaker, Robert M Lawless and Charles J Tabb (eds), *A Debtor World: Interdisciplinary Perspectives on Debt* (OUP 2012) 295–8.

36 Akerlof and Shiller (n 12) x, xii–xvi.

37 Hugh Collins, ‘Oren Bar-Gill, Seduction by Contract: Law, Economics, and Psychology in Consumer Markets’ (2014) 77 MLR 1030, 1034.

38 Federal Trade Commission, ‘Negative Option Rule: A Proposed Rule by the Federal Trade Commission on 04/24/2023’ (2023) 88 Federal Register 24716.

39 In the UK, the FCA has found that 80% of consumers do not consider interest rates when shopping for credit cards, and are more influenced by promotional offers. Of consumers who specifically do not intend to use their credit cards for borrowing, approximately 20% will do so within a year of taking out the product: FCA, ‘Credit Card Market Study: Interim Report’ (2015) MS14/6.2, 6, 41.

40 ibid 12.

41 Ronald J Mann, ‘Bankruptcy Reform and the Sweat Box of Credit Card Debt’ (2007) 2007 U Ill L Rev 375.

42 In 2017, 30% of the £2.4bn in overdraft revenue earned by banks came from unarranged overdrafts, despite representing only 4% of overdraft lending: FCA, ‘High-Cost Credit Review: Overdrafts Consultation Paper and Policy Statement’ (2018) Consultation Paper CP18/42. Overdraft revenues have fallen since the FCA took significant regulatory action in 2019—revenues had reached average levels of approximately £1.6bn per year over the period from mid-2018 to 2020, and had fallen to an average rate of £0.9bn per year by late 2021. FCA, ‘An Evaluation of Our 2019 Overdrafts Intervention’ (2023) Evaluation Paper 23/1, 40.

43 FCA, ‘Overdrafts CP18/42’ (n 42) 3, 15.

44 FCA, ‘High-Cost Credit Review—Overdrafts’ (2018) Consultation Paper CP18/13, 10, 20–1.

45 FCA, ‘Overdrafts CP18/42’ (n 42) 18–19.

46 Sarah Beddows and Mick McAteer, ‘Payday Lending: Fixing a Broken Market’ (Association of Chartered Certified Accountants 2014) 21.

47 Office of Fair Trading, *Payday Lending: Compliance Review Final Report* (OFT 2013) 9.

48 FCA, ‘Proposals for a Price Cap on High-Cost Short-Term Credit’ (2014) Consultation Paper CP14/10, 44.

49 Beddows and McAteer (n 46) 21; the market grew from £900m to £2.2bn from 2008/9 to 2011/12; FCA, ‘Detailed Proposals for the FCA Regime for Consumer Credit’ (2013) Consultation Paper CP13/10, 52. See generally Jodi Gardner, *The Future of High-Cost Credit: Rethinking Payday Lending* (Hart Publishing 2022).

50 Alan M White, ‘Behavior and Contract’ (2009) 27 Law and Inequality 135, 159, 161–2.

51 Tom Baker and Peter Siegelman, ‘“You Want Insurance with That?” Using Behavioral Economics to Protect Consumers from Add-on Insurance Products’ (2013) 20 Conn Insur LJ 1.

52 Rasmus Fleischer, ‘Universal Spotification? The Shifting Meanings of “Spotify” as a Model for the Media Industries’ (2021) 19 Popular Communication 14; firms increasingly blend products with services, such that even purchases of physical items now involve longer-term complex contracts: Chris Jay Hoofnagle, Aniket Kesari and Aaron Perzanowski (eds), ‘The Tethered Economy’ (2019) 87 Geo Wash L Rev 783.

53 Margaret Jane Radin, ‘Form Contracts and the Problem of Consumer Information: Comment’ (2011) 167 Journal of Institutional and Theoretical Economics 49, 51–3.

54 James R Hackney, ‘Law and Neoclassical Economics Theory: A Critical History of the Distribution/Efficiency Debate’ (2003) 32 Journal of Socio-Economics 361.

55 Even those concerned with macroeconomic and distributional effects of retail markets tend to adopt ‘siloed’ approaches, focusing on particular markets or sectors: Rory Van Loo, ‘Broadening Consumer Law: Competition, Protection, and Distribution’ (2019) 95 Notre Dame L Rev 211.

56 Louis Kaplow and Steven Shavell, ‘Why the Legal System Is Less Efficient than the Income Tax in Redistributing Income’ (1994) 23 JLS 667, 674.

57 For recent discussions of this perspective, see eg Raghavan (n 26); Caroline Cecot, ‘Efficiency and Equity in Regulation’ (2023) 76 Vand L Rev 361.

58 Armstrong and Vickers (n 14).

59 David Caplovitz, *Poor Pay More: Consumer Practices of Low Income Families* (Free Press 1968).

60 Sara Davies and Andrea Finney, ‘The Poverty Premium and Debt’ in Jodi Gardner, Mia Gray and Katharina Moser (eds), *Debt and Austerity* (Edward Elgar Publishing 2020).

61 FCA, ‘Overdrafts CP18/42’ (n 42) 3.

62 FCA, ‘Overdrafts CP18/13’ (n 44) 14.

63 Sendhil Mullainathan and Eldar Shafir, *Scarcity: Why Having Too Little Means So Much* (Allen Lane 2013).

64 Melinda Cooper, ‘Money as Punishment: Neoliberal Budgetary Politics and the Fine’ (2018) 33 Australian Feminist Studies 187, 199–200.

65 Desmond (n 9) 74; Davies and Finney (n 60) 184.

66 Lawrence M Ausubel, ‘The Failure of Competition in the Credit Card Market’ (1991) 81 American Economic Review 50.

67 See eg Kai Ruggeri and others, ‘The Persistence of Cognitive Biases in Financial Decisions across Economic Groups’ (2023) 13 Scientific Reports 10329.

68 Sumit Agarwal and others, ‘Regulating Consumer Financial Products: Evidence from Credit Cards’ (2015) 130 Quarterly Journal of Economics 111.

69 Stiglitz (n 1) 37.

70 FCA, ‘Monthly PPI Refunds and Compensation’ (29 April 2021) <www.fca.org.uk/data/monthly-ppi-refunds-and-compensation> accessed 14 September 2022.

71 The FCA estimates that its 2019 regulatory measures in the overdraft market has saved consumers about £500m per year: FCA, ‘Overdrafts Evaluation Paper 23/1’ (n 42) 40. The FCA estimated that previous measures would save credit card users £310m–£1.3b annually: FCA, ‘Credit Card Market Study: Consultation on Persistent Debt and Earlier Intervention Remedies’ (2017) Consultation Paper CP17/10 10. See also Competition and Markets Authority, ‘Tackling the Loyalty Penalty: Response to a Super-Complaint Made by Citizens Advice on 28 September 2018’ (2018) (recognising a £4bn/year ‘loyalty penalty’ across five retail markets); Will Dobbie and Jae Song, ‘Debt Relief and Debtor Outcomes: Measuring the Effects of Consumer Bankruptcy Protection’ (2015) 105 American Economic Review 1272 (highlighting how the US bankruptcy system redistributes $450bn/year to consumers, exceeding the amount transferred through all US state employment insurance); Van Loo (n 55) (estimating that annual overcharging in US consumer markets exceeds $1tn/year).

72 Fred Shapiro and Michelle Pearse, ‘The Most-Cited Law Review Articles of All Time’ (2012) 110 Mich L Rev 1483.

73 RH Coase, ‘The Problem of Social Cost’ (1960) 3 JLE 1.

74 Even if this support often involves a mistaken understanding of some of the piece’s key points: Guido Calabresi, ‘The Pointlessness of Pareto: Carrying Coase Further’ (1991) 100 Yale LJ 1211, 1213; John Cassidy, ‘Ronald Coase and the Misuse of Economics’ *The New Yorker* (3 September 2013).

75 Robert L Hale, ‘Bargaining, Duress, and Economic Liberty’ (1943) 43 Colum L Rev 603, 625.

76 Robert H Mnookin and Lewis Kornhauser, ‘Bargaining in the Shadow of the Law: The Case of Divorce’ (1979) 88 Yale LJ 950.

77 Eric Posner and E Weyl, *Radical Markets: Uprooting Capitalism and Democracy for a Just Society* (Princeton UP 2018) ch 1.

78 David Campbell, ‘The Fetishism of Divergence: A Critique of Piketty’ (2015) 15 JCLS 183, 212–13.

79 Stiglitz (n 1) 193; Robert B Reich, *Saving Capitalism: For the Many, Not the Few* (Knopf Publishing Group 2015) ch 7.

80 Eric A Posner and Cass R Sunstein, ‘Antitrust and Inequality’ (2022) 2 American Journal of Law and Equality 190.

81 Katharina Pistor, *The Code of Capital* (Princeton UP 2019) 4.

82 Yair Listokin, *Law and Macroeconomics* (Harvard UP 2019); Spooner (n 33) ch 3; Atif Mian and Amir Sufi, ‘The Macroeconomic Advantages of Softening Debt Contracts’ (2017) 11 Harvard Law and Policy Review 11.

83 Kaplow and Shavell (n 56).

84 Listokin (n 82) 3.

85 Duncan Kennedy, ‘The Stakes of Law, or Hale and Foucault Transformative Discourses in Postmodern Social Cultural and Legal Theory’ (1991) 15 Legal Studies Forum 327, 334.

86 For example, approaches theorising contract as promise tend towards a ‘myopic’ scope which focuses only on commercial contracts: Prince Saprai, ‘Moving Beyond Promise: A Response to The Choice Theory of Contracts’ (2019) 20 JRLS 104. For a recent re-theorisation of contract which rejects distributional concerns, see Peter Benson, *Justice in Transactions: A Theory of Contract Law* (1st edn, Belknap Press 2020).

87 Ramsay, ‘Consumer Credit’ (n *) 185; Kennedy, ‘Stakes’ (n 85) 350.

88 Jodi Gardner, ‘Being Conscious of Unconscionability in Modern Times: Heller v Uber Technologies’ (2021) 84 MLR 874, 883–5.

89
*Payward, Inc & Ors v Chechetkin* [2023] EWHC 1780 (Comm) [76]; see also *Soleymani v Nifty Gateway LLC* [2022] EWCA Civ 1297 (6 October 2022).

90 Collins, ‘Review of Bar-Gill’ (n 38) 1033.

91 FCA, ‘High-Cost Credit Review: Feedback on CP18/12 with Final Rules and Guidance and Consultation on Buy Now Pay Later Offers’ (2018) Consultation Paper CP18/43, 41; HM Treasury, ‘Regulation of Buy-Now Pay-Later: Consultation’ (2021). A key theme across these regulatory reports is the question of the appropriate legal categorisation and consequent regulatory regime for new buy-now pay-later products.

92 Ramsay, ‘Consumer Credit’ (n *) 185.

93 Kennedy, ‘Stakes’ (n 85) 348–9.

94 Ramsay, ‘Consumer Credit’ (n *) 184.

95 Hugh Collins, ‘Interpersonal Justice as Partial Justice’ (2022) 1 European Law Open 413, 417; Iain Ramsay, ‘Productive Disintegration and the Law of Contract’ (2004) 2004 Wis L Rev 495.

96 Nick Sage, ‘On Justice in Transactions’ (2021) 84 MLR 898.

97 Hugh Collins, ‘Utility and Rights in Common Law Reasoning: Rebalancing Private Law Through Constitutionalization’ (2007) 30 Dalhousie Law Journal 1; Aditi Bagchi, ‘Distributive Justice and Contract’ in Gregory Klass, George Letsas and Prince Saprai (eds), *Philosophical Foundations of Contract Law* (OUP 2014).

98 Sage (n 96).

99 Collins, ‘Utility’ (n 97).

100 Stewart Macaulay, ‘Private Legislation and the Duty to Read—Business Run by IBM Machine, the Law of Contracts and Credit Cards’ (1965) 19 Vand L Rev 1051, 1076.

101 Susan Block-Lieb and Edward J Janger, ‘Fit for Its Ordinary Purpose: Implied Warranties and Common Law Duties for Consumer Finance Contracts’ (2021) 59 Hous LR 3.

102 FCA, ‘CP14/10’ (n 48).

103 Michelle Kelly-Louw, ‘The Statutory in Duplum Rule as an Indirect Debt Relief Mechanism’ (2011) 23 South African Mercantile Law Journal 352.

104
*ParkingEye* (n 15). What follows is an intentionally deconstructed account of key features of this decision, adopting a thematic analysis of key points made by the court regarding the penalty doctrine and unfair terms. For more direct expert exposition of the Supreme Court judgments, see eg Andrew Summers, ‘Unresolved Issues in the Law on Penalties’ [2017] LMCLQ 95; Solène Rowan, ‘The “Legitimate Interest in Performance” in the Law on Penalties’ [2019] CLJ 1; Roger Halson, *Liquidated Damages and Penalty Clauses* (UP 2018) ch 2.

105 Kennedy, ‘Stakes’ (n 85) 327–8.

106
*ParkingEye* (n 15) para 42. Lords Neuberger and Sumption referred to both law firms by brand name.

107 In requiring an additional sum of 70% of the initial charge in the event of late payment, this structure effectively piled penalty clause upon alleged penalty clause.

108 From machine readings of Mr Beavis’s car registration, ParkingEye then received Mr Beavis’s data from the Driver and Vehicle Licensing Agency, in a manner emblematic of contemporary ‘surveillance capitalism’: Shoshana Zuboff, *The Age of Surveillance Capitalism* (1st edn, PublicAffairs 2019). Digital surveillance, in turn, provides the main sanction for those who default in meeting a parking charge payment—a CCJ, or County Court Judgment notice, may be entered on a defaulting party’s credit history, impacting an individual’s future access to markets, housing and even employment: see discussion in Spooner (n 33) 261–7.

109 The 1999 Regulations have been superseded by Part II of the Consumer Rights Act 2015. Both are based on European Union legislation: EU Council Directive 93/13/EEC on unfair terms in consumer contracts 1993.

110 For the provision currently in force, see Consumer Rights Act 2015, Sch 2, Part 1(6).

111 Note the intervention of the Consumers’ Association and the public commentary on the case by the British Parking Association: ‘Barry Beavis’s Supreme Court Parking Appeal Cash Bid Reached’ (*BBC News*, 29 April 2015) <www.bbc.com/news/uk-england-essex-32509849> accessed 30 September 2022.

112 Claire Brader, ‘Private Parking Code of Practice: Latest Developments’ [2023] In Focus: House of Lords Library.

113 Its accounts at Companies House show revenues of almost £50m in 2022, with gross profit margins of approximately 62.5%.

114 Brett Christophers, *Our Lives in Their Portfolios: Why Asset Managers Own the World* (Verso 2023) 13.

115 Iain Ramsay, ‘Assembly-Line Debt Collection and the International Overindebtedness Industry’ (*creditdebtandinsolvency*, 29 July 2022) <https://creditdebtandinsolvency.wordpress.com/2022/07/29/assembly-line-debt-collection-and-the-international-overindebtedness-industry/> accessed 30 September 2022.

116 Daniel Wilf-Townsend, ‘Assembly-Line Plaintiffs’ (2022) 135 Harv L Rev 1704.

117 Debt claims make up 86% of County Court claims, of which 90% are undefended. If we narrow analysis to claims under £1000, the number of undefended claims rises to 94%: Linda Mulcahy and Wendy Teeder, ‘Are Litigants, Trials and Precedents Vanishing After All?’ (2022) 85 MLR 326. Much work of the courts now appears to be the production of CCJ data inputs for an expansive credit reporting industry: Spooner (n 33) 261–7.

118 Sir Geoffrey Vos, ‘The Proper Place of Law in a Digital Society’ (Speech by the Master of the Rolls to the Socio-Legal Studies Association Conference 2022) <www.judiciary.uk/speech-by-the-master-of-the-rolls-to-the-socio-legal-studies-association-conference/> accessed 13 February 2023. See discussion in Kate Leader, ‘The Small Claims Paper Determination Pilot: Filtering out the County Courts’ “Garbage Claims”’ (*The Modern Law Review Forum*, 2022) <www.modernlawreview.co.uk/leader-small-claims-pilot/> accessed 25 June 2022.

119 Lord Mance noted that the ‘cases lie at opposite ends of a financial spectrum’: *ParkingEye* (n 15) para 116; meanwhile, a commentator contrasted the loss of £44m at stake in the *Makdessi* case with a case involving ‘a low value consumer contract’, bearing the only consequence of the payment of an £85 fee: Halson (n 104) para 4.71. Neither comment recognises that the stakes in the *ParkingEye* decision, in laying ground rules to regulate what appears to be a billion-pound market, vastly outweighed the *Makdessi* case in monetary terms.

120 The US Supreme Court has also recently decided a case based upon a parking ticket debt: *City of Chicago, Illinois v Fulton* 592 US__ (2021) (Supreme Court of the United States); this case arose as ‘part of a nationwide trend in which municipalities rely heavily on fines, fees, and punitive collection practices for revenue’, and a context in which Chicago residents owe $1.45 billion in unpaid parking tickets: American Civil Liberties Union and others, ‘City of Chicago, Illinois v Fulton, Brief of the American Civil Liberties Union et al. as Amici Curiae in Support of the Respondents’ 3.

121 See eg Rowan (n 104).

122 Bagchi (n 97) 193; Margaret Jane Radin, *Boilerplate: The Fine Print, Vanishing Rights, and the Rule of Law* (Princeton UP 2012) 3–18.

123 Stewart Macaulay, ‘Non-Contractual Relations in Business: A Preliminary Study’ (1963) 28 American Sociological Review 55.

124 Arthur Allen Leff, ‘Injury, Ignorance and Spite—The Dynamics of Coercive Collection’ (1970) 80 Yale LJ 1.

125 Collins, ‘Interpersonal Justice’ (n 95).

126 For discussion of this viewpoint in relevant literature, see Bagchi (n 97) 193; for a key example of this approach, see Kaplow and Shavell (n 56).

127 These conversations also ignore the reality that while private law has not typically based liability rules on individual parties’ income, it commonly designs discrete rules for disadvantaged classes or groups—most notably in consumer and employment law: Kennedy, ‘Stakes’ (n 85).

128 Corinne Blalock, ‘Neoliberalism and the Crisis of Legal Theory Law and Neoliberalism’ (2014) 77 LCP 71, 77.

129 Laura Nader, ‘A User Theory of Law’ (1984) 38 SMU L Rev 951, 960.

130
*ParkingEye* (n 15) paras 12–14.

131 Summers (n 104).

132
*ParkingEye* (n 15) paras 40, 43, 130, 238.

133
*Abbey National* (n 16) para 83.

134 See what is now Consumer Rights Act 2015, s 64.

135 The first instance decision in *Abbey National* had already found that the penalty rule did not apply to overdraft fees, as technically the contingency leading to these charges becoming payable was not a breach of contract: *Office of Fair Trading v Abbey National plc and Others* [2008] EWHC 875 (Comm) [295]–[307].

136
*Abbey National* (n 16) para 47.

137 The Court also accepted a second argument by Mr Sumption that unauthorised overdrafts constitute an ‘extremely valuable facility’ allowing informal overdrafts ‘at short notice and without elaborate negotiation’: ibid 85. Again, this reasoning appears detached from regulatory analysis of the market for unarranged overdrafts, in which a primary consumer harm is the disproportionately high cost of such products compared to other available facilities. As noted above, in half of cases, an unarranged overdraft is of negative financial value.

138
*Andrews v Australia and New Zealand Banking Group Limited* [2012] HCA 30.

139 ibid 5.

140
*ParkingEye* (n 15) paras 3, 42–3.

141 Some criticisms of the penalty doctrine tend to assume the existence of ‘free agreement’, and so assume away central complexities of contemporary contracting: Jonathan Morgan, ‘The Penalty Clause Doctrine: Unlovable but Untouchable’ (2016) 75 CLJ 11, 12, 14; for a nuanced consideration of contractual freedom in modern markets, see Gardner (n 49) ch 3.

142 Margaret Jane Radin, ‘An Analytical Framework for Legal Evaluation of Boilerplate’ in Gregory Klass, George Letsas and Prince Saprai (eds), *Philosophical Foundations of Contract Law* (OUP 2014) 215.

143
*ParkingEye* (n 15) para 108.

144 Drawing on the application of the relevant European unfair terms legislation by the Court of Justice, Lords Neuberger and Sumption explained that the legislative test of whether a term created a ‘significant imbalance’, ‘contrary to the requirement of good faith’, would depend on whether a firm could ‘reasonably assume that the consumer would have agreed to such a term in a negotiation’: ibid 105–9, citing Case C-41511 *Mohamed Aziz v Catalunyacaixa* [2013] [69].

145
*ParkingEye* (n 15) para 109.

146
*The Office of Fair Trading v Abbey National plc & Others* [2009] EWCA Civ 116 [104]–[109].

147
*Abbey National* (n 16) para 79.

148 Michael Schillig, ‘Inequality of Bargaining Power Versus Market for Lemons: Legal Paradigm Change and the Court of Justice’s Jurisprudence on Directive 93/13 on Unfair Contract Terms’ [2008] EL Rev 336.

149 Bar-Gill, *Seduction by Contract* (n 11).

150
*Abbey National* (n 16) para 80.

151 Blalock (n 128) 99.

152 Macaulay, ‘Private Legislation’ (n 100) 1085.

153 Duncan Kennedy, ‘The Bitter Ironies of Williams v Walker-Thomas Furniture Co. In the First Year Law School Curriculum’ (2023) 71 Buff L Rev 225, 234–5.

154 For an example, see Yeşim M Atamer, ‘Why Judicial Control of Price Terms in Consumer Contracts Might Not Always Be the Right Answer—Insights from Behavioural Law and Economics’ (2017) 80 MLR 624; Ramsay illustrates how similar reasoning dominated in a market-shaping House of Lords decision one century ago: Iain Ramsay, ‘Helby v Matthews: The ‘Great Test Case’?’ in Jodi Gardner and Iain Ramsay (eds), *Landmark Cases in Consumer Law* (Hart Publishing 2023).

155
*Abbey National* (n 16) paras 1–3.

156 Kennedy, ‘Merely Technical’ (n 18).

157
*Abbey National* (n 16) para 93.

158
*Dunlop Pneumatic Tyre Co Ltd v New Garage & Motor Co Ltd* [1914] UKHL 1.

159 Halson (n 104) paras 2.07, 2.38; criticisms by contract minimalists of the ‘timid’ failure of the Court to abolish the penalty doctrine outright thus seem founded more in form than substance: Morgan (n 141); on which, see Duncan Kennedy, ‘Form and Substance in Private Law Adjudication’ (1975) 89 Harv L Rev 1685.

160
*ParkingEye* (n 15) para 32.

161 ibid 104.

162 ibid 103–5.

163 ibid 97.

164 For a related discussion of remedial clauses which emphasises the traditional judicial functions involved in determining appropriate remedies, see Seana Shiffrin, ‘Remedial Clauses: The Over-Privatization of Private Law’ (2016) 67 Hastings LJ 407.

165 Campbell and Halson explain that this involved ‘something which had never before been done in the common law of contract’, in sanctifying the prevention of breach irrespective of the adequacy of damages: David Campbell and Roger Halson, ‘By Their Fruits Shall Ye Know Them’ (2020) 79 CLJ 405, 407.

166
*ParkingEye* (n 15) para 99.

167 ibid 98.

168 ibid 107.

169 ibid 98.

170 Ramsay, ‘Consumer Credit’ (n *) 184–5; Duncan Kennedy, *A Critique of Adjudication [Fin de Siecle]* (Harvard UP 1998) 149.

171 Collins, ‘Utility’ (n 97) 4.

172
*Dunlop* (n 158).

173 Lords Neuberger and Sumption acknowledge the illegality of resale price maintenance, but seem reassured by the idea that it ‘was a legitimate restriction of competition according to the notions prevailing in 1914’: *ParkingEye* (n 15) para 21.

174
*Paciocco v Australia and New Zealand Banking Group Limited* 2016 HCA 28 (High Court of Australia).

175
*ParkingEye* (n 15) para 195.

176 Here, the court found that a gym firm’s standard form contract contained a ‘trap’, and its ‘business model [was] designed and calculated to take advantage of the naivety and inexperience of the average consumer’: *Office of Fair Trading v Ashbourne Management Services Ltd* [2011] EWHC 1237 (Ch) [173].

177 The rejection of this analysis as ‘fact specific’ ignores findings of behavioural economics literature that these pricing practices are replicated throughout the economy: Akerlof and Shiller (n 12); Bar-Gill, *Seduction by Contract* (n 11).

178
*ParkingEye* (n 15) para 211.

179 In dissent, Lord Toulson accepts the ‘telling points’ made by the Consumers’ Association, highlighting how unforeseen external circumstances may prevent a consumer from leaving within two hours: ibid 310.

180 ibid 109.

181 ibid 109, 113.

182
*Abbey National* (n 16) para 2.

183 Blalock (n 128) 100.

184
*Irish Bank Resolution Corporation Ltd v Cambourne Investments Inc & Ors* [2012] IEHC 262 [44].

185 Macaulay, ‘Private Legislation’ (n 100) 1058.

186
*ParkingEye* (n 15) para 31.

187 ibid 111.

188
*Aziz* (n 144) para AG87.

189 When an asset manager purchases a piece of infrastructure such as car parking facilities, ‘the first and most obvious rule is simply to maximise revenues’. Christophers (n 114) 219.

190 Substantial incentives already exist to encourage debt repayment, whether from credit reporting systems or moral obligations: Spooner (n 33) 261–7; Francesca Polletta and Zaibu Tufail, ‘The Moral Obligations of Some Debts’ (2014) 29 Sociological Forum 1; Pamela Foohey and others, ‘Life in the Sweatbox’ (2018) 94 Notre Dame L Rev 219.

191
*ParkingEye* (n 15) para 97.

192 ibid 23, 29, 32.

193
*Ashbourne* (n 176) para 173.

194 Morgan (n 141) 13–14; *ParkingEye* (n 15) para 3.

195
*ParkingEye* (n 15) para 109.

196 ibid 100.

197 Joseph Spooner, ‘The Local Austere Creditor’ in Saul Schwartz (ed), *Oppressed by Debt: Government and the Justice System as a Creditor of the Poor* (Routledge 2022) 42.

198 Cooper (n 64).

199 RAC Foundation, ‘Council Parking Revenue in England 2018–19’ (16 November 2019) <www.racfoundation.org/research/mobility/council-parking-revenue-in-england-2018-19> accessed 28 September 2020.

200 House of Commons Treasury Committee, *Household Finances: Income, Saving and Debt*’ (2018, HC 565) 16.

201 Money Advice Trust, ‘Stop the Knock: An Update on Local Authority Debt Collection Practices in England and Wales’ (2019).

202 American Civil Liberties Union and others (n 120) 16–17; Christophers (n 114) 220.

203 Akerlof and Shiller (n 12). For example, the scandal of the PPI episode was not that one firm engaged in abusive practices, but that the entire UK banking industry systematically mis-sold PPI products at great profit.

204
*Paciocco* (n 174) para 290.

205 FCA, ‘Overdrafts CP18/42’ (n 42) 15.

206 Lord Toulson’s dissent demonstrates how such an approach could have worked under unfair terms legislation, when the default charge could be considered unfair due to the way it greatly exceeds the consumer’s (nominal) legal liability in the absence of the clause: *ParkingEye* (n 15) para 307.

207
www.ce-service.co.uk/.

208 For discussion of how courts can legitimate market practices in this way, see Ramsay, ‘Helby’ (n 154).

209 Campbell and Halson (n 165) 405, citing DDJ Harvey in *One Parking Solution Ltd v W*, 5 February 2020 (Lewes County Court).

210 Brader (n 112).

211 The FSA PPI redress scheme, and the £50bn it redistributed into the UK economy, itself had to survive court approval in a judicial review challenge: *R (British Bankers’ Association) v The Financial Services Authority and the Financial Ombudsman Service* [2011] EWHC 999 (Admin).

212
*Abbey National* (n 16).

213 FCA, ‘Overdrafts CP18/42’ (n 42); the FCA estimates that its measures have saved consumers £500m per year: FCA, ‘Overdrafts Evaluation Paper 23/1’ (n 42) 40.

214 Macaulay, ‘Private Legislation’ (n 100) 1069.

215 For contrast, see the discussion of Professor Hesselink’s agenda in Collins, ‘Interpersonal Justice’ (n 95).

216 Kennedy, ‘Stakes’ (n 85) 348–9.

217 JAG Griffith, *Politics of the Judiciary* (5th edn (reissue), Fontana Press 2010) 293.

218 Macaulay, ‘Private Legislation’ (n 100) 1069.

219 PS Atiyah, ‘Freedom of Contract and the New Right’, *Essays on Contract* (OUP 1986) 363.

220 ibid.

221 Lee Epstein, William M Landes and Richard A Posner, ‘When It Comes to Business, the Right and Left Sides of the Court Agree Judicial Behavior of the Roberts Court’ (2017) 54 Washington University Journal of Law and Policy 33; Wendy Brown, *Undoing the Demos: Neoliberalism’s Stealth Revolution* (MIT Press 2015) ch 5; Jedediah Purdy, ‘Neoliberal Constitutionalism: Lochnerism for a New Economy Law and Neoliberalism’ (2014) 77 LCP 195.

222 Chris Hanretty, ‘Haves and Have-Nots before the Law Lords’ (2014) 62 Political Studies 686, 686.

223 Ramsay, ‘Consumer Credit’ (n *) 183.

224 Dan Priel, ‘The Law and Politics of Unjust Enrichment’ (2013) 63 UTLJ 533, 548, 549–53; see Duxbury’s insight that UK legal theory has lacked a counterpart to the ‘radical legal thought’ and ‘doctrinal legal critique’ developed by Robert Hale and other authors associated with legal realism: Neil Duxbury, ‘Robert Hale and the Economy of Legal Force’ (1990) 53 MLR 421, 421, 444.

225 Brown (n 221) 21; see also Elizabeth Popp Berman, *Thinking Like an Economist: How Efficiency Replaced Equality in US Public Policy* (Princeton UP 2022).

226 Colin Crouch, *Can Neoliberalism Be Saved From Itself?* (Social Europe Edition 2017).

227 James Kwak, *Economism: Bad Economics and the Rise of Inequality* (Pantheon Books 2017).

228 Crouch (n 226) 19.

229 See eg Joseph E Stiglitz, ‘Is There a Post-Washington Consensus Consensus?’ in Narcís Serra (ed), *The Washington Consensus Reconsidered: Towards a New Global Governance* (OUP 2008).

230 Lord Toulson’s words: *ParkingEye* (n 15) para 315.

231 Kennedy, *Critique* (n 170) 149.

